# Evaluation of multiple satellite precipitation products and their potential utilities in the Yarlung Zangbo River Basin

**DOI:** 10.1038/s41598-022-17551-y

**Published:** 2022-08-03

**Authors:** Haoyu Ji, Dingzhi Peng, Yu Gu, Yaqi Liang, Xiaoyu Luo

**Affiliations:** 1 College of Water Sciences, Beijing Normal University, Beijing, 100875 China; 2 Beijing Key Laboratory of Urban Hydrological Cycle and Sponge City Technology, Beijing, 100875 China

**Keywords:** Climate sciences, Hydrology

## Abstract

Hydrological modeling in the Third Pole remains challenging due to the complex topography and scarcity of in-situ precipitation observations. In this study, we assessed five satellite precipitation products (SPPs) including TRMM3B42, PERSIANN-CDR, GPM-IMERG, CMORPH, and GSMaP, and simulated daily streamflow in the Yarlung Zangbo River Basin (YZRB) with VIC model. The performance of SPPs was evaluated by *CC*, *RB*, *RMSE*, *POD* and *FAR*, to compare with daily observations. Overall, all SPPs showed decreasing trends of precipitation from east to west compared to 10 km rainfall data. PERSIANN had the highest values of *POD* (0.65), *RB* (91.6%) and *FAR* (0.59) but worst performed in streamflow. CMORPH, GPM and TRMM fit well with the observations annually but overestimate the precipitation in the southeast during wet seasons. Simulation from GPM and CMORPH yield satisfactory results (*NSE* of 0.86 and 0.82, *RE* of − 20% and − 13%, respectively), while TRMM outperformed GPM in modeling runoff with smaller relative error. Results indicated the potential of GPM and CMORPH in providing alternative rainfall information in YZRB. Accurate evaluation of multi-source SPPs and their hydrological utility in YZRB would benefit further hydrometeorological studies and water resources management in this area.

## Introduction

As a critical factor in the atmosphere cycle, precipitation drives the hydrological cycle and influences the energy cycle. There are three main ways to measure precipitation events: observed gauges, radar, and satellite. Gauged observation is the traditional approach to obtaining accurate precipitation estimations at a given point. Due to the complex topography, precipitation and its spatial variability are irregular and unavailable in the watershed with sparse gauges^1^. However, the occurrence of satellite deployed PR-related infrared and microwave satellite sensors provides a unique opportunity for precipitation estimation from the gridded scale. Despite various errors and uncertainties, satellite precipitation products (SPPs) have become essential sources of precipitation information, especially in regions where the gauged distribution is sparse and uneven^2^. Currently, SPPs have been widely used in water resources management^3,4^, drought monitoring^5–7^, and flood forecasting^8,9^.

In the twentieth century, techniques of SPPs with different temporal and spatial resolutions had achieved increasing maturity^10^, such as Tropical Rainfall Measurement Mission (TRMM)^11^, Precipitation Estimation from Remotely Sensed Information using Artificial Neural Networks (PERSIANN)^12^, National Oceanic and Atmosphere Administration/Climate Prediction Center morphing technology (CMORPH)^13,14^, Global Precipitation Measurement (GPM) and Global Satellite Mapping of Precipitation (GSMaP)^15^. Most SPPs had a good correspondence with gauged estimation since the gauge information is integrated with the correction algorithm^2,16,17^. Purely satellite-based estimation without any gauged corrections tended to overestimate gauged observation, primarily due to the weak relationship between rainfall rate and remote sensing signal, sampling uncertainties together with error caused by human algorithms or atmospheric environmental effects^18–22^. Furthermore, the errors in the SPPs can be propagated and expanded in the hydrological utility due to the nonlinearities in the hydrological process^17^. Therefore, the accurate assessment of SPPs is an indispensable part of their application in both hydrology and meteorology.

There were two types of validation methods for SPPs: (1) directly statistical metrics of satellite precipitation against the corresponding gauged observation or the weather radar estimation; and (2) evaluation of the satellited precipitation based on a model frame^23^. Numerous validation researches on SPPs have been carried out to better understand the uncertainties of different products over different regions^24–31^. Because of the unique topography and profound impact on regional climate and even on a global scale^32^, most of the corresponding researches on SPPs have focused on the Qinghai Tibet Plateau (TP)^2,16,33^. For example, Gao and Liu^16^ compared TRMM3B42, TRMM3B42-RT, CMORPH, and PERSIANN against gauged observation and found that the four SPPs tended to overestimate light rainfall and underestimate moderate and heavy rainfall while performing better in the humid regions than arid regions. Lu and Yong^33^ evaluated the capacities of GPM and GSMaP in rainfall detection and found inaccurate records of light rain and snow. Some researchers have devoted their efforts to assessing the suitability of SPPs as input to hydrological models in the TP^34^. Tong and Su^2^ investigated the capability of four SPPs (CMORPH, PERSIANN, TRMM3B42, and TRMM3B42-RT) by using a Variable Infiltration Capacity (VIC) model and found that CMORPH and TRMM3B42 performed better in hydrological utilities than TRMM3B42RT and PERSIANN. Similarly, Wu and Guo^35^ found SPPs performed better in the southeast of the TP and pointed out that China Meteorological Forcing Dataset (CMFD) could yield higher accuracy than TRMM and CHIRPS in streamflow simulation.

Yarlung Zangbo River Basin (YZRB), characterized by the strong influence of the monsoon, differs from the rest of the TP in hydrologic regimes. Like other areas in TP, the sparsely distributed rainfall gauges in the YZRB result in a lack of in-situ observations and call for the assessment of alternative precipitation data. Luo and Fan^36^ validated the hydrological potential of APHRODITE in the whole Yarlung Zangbo-Brahmaputra River basin and made a trial to improve the accuracy through linear correction. The results revealed that APHRODITE systematically underestimated precipitation in the rainy season. Liu and Xu^37^ assessed the accuracy of Precipitation Estimation from Remotely Sensed Information using Artificial Neural Networks-Climate Data Record (PERSIANN-CDR) in the YZRB and found that the accuracy decreased from east to the west in the basin. Currently, satellite-based precipitation research in YZRB mainly focused on the correction and evaluation of a single remote sensed product. As popular and important as individual SPP might be in YZRB, comprehensive and comparative assessments of multi-satellite products are relatively few, mainly due to limited rainfall data at gauges^36,37^. It is therefore useful to evaluate how different the potentials of various SPPs are in estimating precipitation in YZRB..

Reconstructed daily precipitation datasets with high precision may benefit hydrological simulation and SPP evaluation^38,39^. In this study, a 10 × 10 km reconstructed precipitation dataset that is based on 262 rain gauges^39^ was introduced to provide gridded rainfall information in the streamflow simulation of YZRB. The primary objective of the study is to evaluate and compare five typical SPPs (CMORPH, TRMM3B42, PERSIANN-CDR, GPM-IMERGE, and GSMaP) in the YZRB. Specifically, the SPPs are (1) compared to gauged observations via diverse statistical metrics (*CC, RB, RMSE, POD, FAR*), then (2) used as forcing datasets to drive the VIC model for runoff simulation through two scenarios. The results would be quite supportive in assessing SPPs over YZRB and provide feedback on SPPs’ hydrological utility in this area, as well as benefit water resources management in ungauged basins.

## Study area and data

### Study area

**Figure 1:**
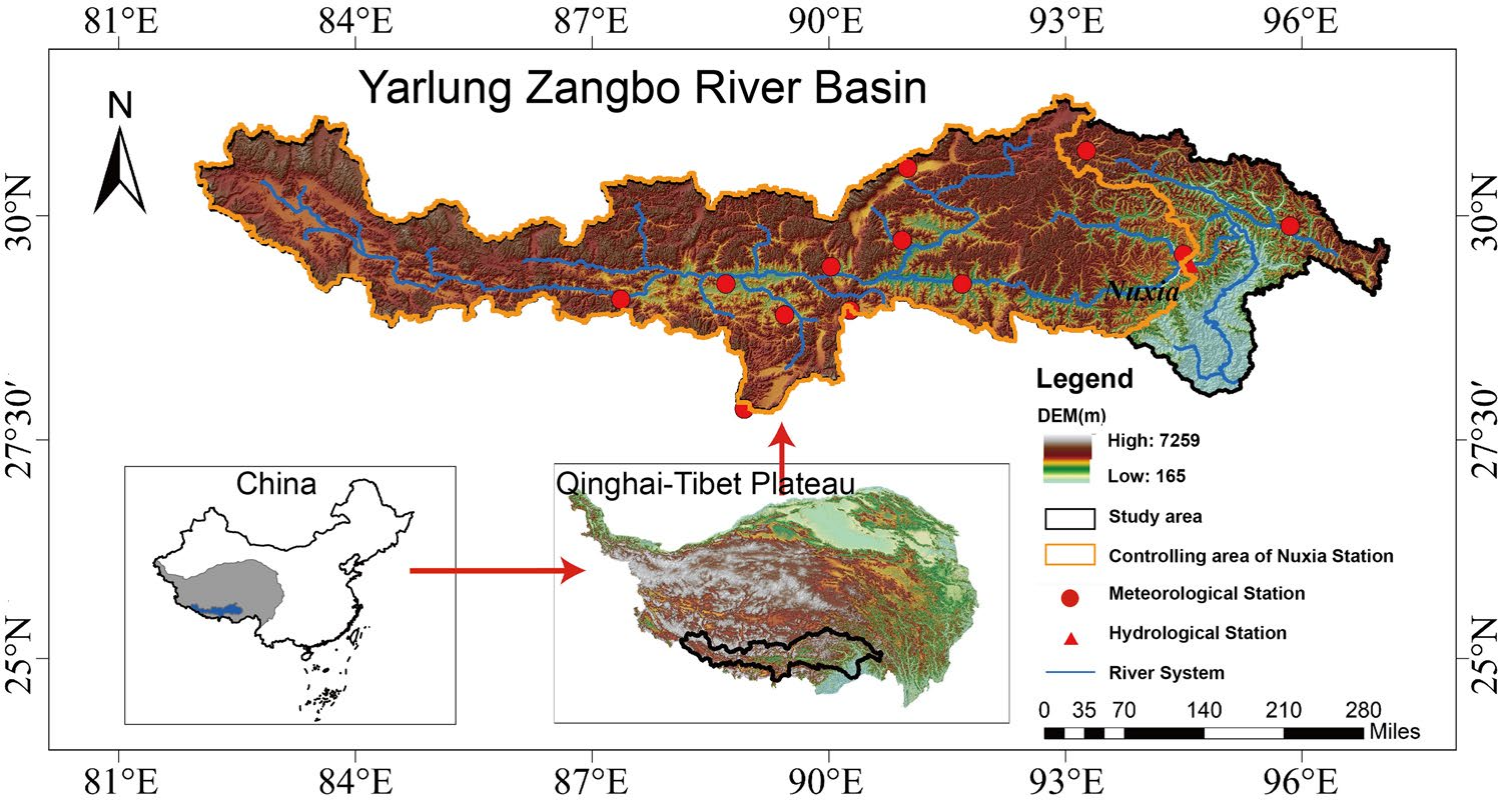
Location, topography and distribution of observation stations in YZRB (The software used to create the maps in Fig. 1, 2, 3, 4, 5 is ArcGIS10.4, http://gisserver.domain.com:6080/arcgis).

The Yarlung Zangbo River is located between 82° ~ 97° 7′ E and 28° ~ 31° 16′ N in the southeast of the TP (Fig. 1). The basin covers an area of 242 000 km^2^, with a river length of 1500 km. The YZRB experiences many different climatic patterns, including alpine temperate semiarid, plateau temperate semiarid, and tropical and subtropical monsoon. The annual average precipitation is 429 mm in the basin, and the spatial distribution of precipitation is quite uneven.

### Data

#### CMORPH

CMORPH, proposed by NOAA (National Oceanic and Atmospheric Administration) Climate Prediction Center via MORPHing technique, provides the precipitation estimation derived from low-orbiter-satellite passive microwave (PMW) observations^13^. The precipitation estimation in CMORPH is generated from radiometers in the satellites (GEOS-8, GOES-10, Meteosat-7, Meteosat-5, and GMS-5). In this study, the 0.25° × 0.25° 3-hourly CMORPH products from 2003 to 2015 were obtained from ftp://ftp.cpc.ncep.noaa.gov/precip/^40^.

#### TRMM 3B42

TMPA is a collaborative product developed by the National Aeronautics and Space Administration (NASA) and Japan Aerospace Exploration Agency (JAXA) based on the calibration of TRMM Combined Instrument and TRMM Microwave Imager precipitation products^11^. TMPA products include two versions. In this study, the 3-hourly TMPA 3B42 V7 with a spatial resolution of 0.25° × 0.25° was applied, which was later referred to as TRMM^41^. The TRMM data in the study are downloaded from http://precip.gsfc.nasa.gov.

#### PERSIANN-CDR

PERSIANN is a product with a spatial resolution of 0.25° and a frequency of 3-h invented by the Center for Hydrometeorology and Remote Sensing (CHRS)^42^. The PERSIANN method^12^ utilizes a neural network function approximation step to convert the IR brightness temperature from a geostationary satellite to precipitation estimation. PERSIANN-CDR differs from the former version in terms of the IR data with the use of GridSat-B1 instead of CPC-IR, and PMWs data is absent in the calibration^43^. In subsequent articles, we will refer to it simply as PERSIANN^44^. The PERSIANN precipitation data are available on http://fire.eng.uci.edu/PERSIANN/.

#### GPM-IMERG

The fine resolution datasets of IMERG (half-hourly at 0.1° × 0.1° grids) is the Level 3 precipitation estimation algorithm of GPM, which provides different products, including an Early Run (near real-time with a latency of 4 h), a Late Run (reprocessed near real-time with a latency of 12 h), and a Final Run (Gauged-adjusted with a latency of 4 months) products. The version used in this study was GPM-IMERG Final Run, which was later referred to as GPM.

#### GSMaP

The GSMaP, an hourly SPP with 0.1° grids resolution, is generated by a program aiming to obtain high precision, high-resolution global precipitation map using satellite data sponsored by the Japan Aerospace Exploration Agency Precipitation Measuring Mission^45^. The GSMaP algorithm utilizes various PMW radiometers to retrieve quantitative precipitation estimation^46^. In this study, the version GSMaP-Gauge^47^ was chosen and applied, which was later referred to as GSMaP.

As GSMsP and GPM-IMERG were finer in spatial coverage, the five satellite-gauge SSPs were first aggregated into the uniform 0.25° × 0.25° spatial resolution and accumulated into daily precipitation amount (00 UTC-00 UTC) during the study period from 2003 to 2015 to match the 8:00 to 8:00 local time of the gauge data in China.

#### Gauged data

The daily observation from 2003 to 2015 in the YZRB was obtained from the China Meteorological Administration (CMA) (http://data.cma.cn), including precipitation, maximum and minimum temperature, and the average wind speed. The daily streamflow data at Nuxia from 2003 to 2015 were primarily collected from the Tibet Hydrology and Water Resources Survey Bureau.

#### Daily gridded precipitation data

The daily gridded precipitation data with the spatial resolution of 10 × 10 km, released by Sun and Su^39^ was adopted as the input data for the VIC model and thus obtaining a set of calibrated model parameters for the subsequent SPPs’ evaluation. The reconstructed data, later referred to as 10 km precipitation data, was generated based on 262 rain gauges and corrected by China Meteorological Administration (CMA) and Global Land Data Assimilation Systems (GLDAS) data, and the datasets had been extensively assessed and validated in some basins^39^. The 10 km precipitation data are obtained from the National Tibetan Plateau Scientific Data Center (http://data.tpdc.ac.cn).

### Methodology

#### Statistical metrics

Two evaluation approaches, the general evaluation via statistical metrics and the detection ability evaluation via categorical metrics, are adopted in assessing the hydrologic skills of the SPPs. The conventional statistical analysis was conducted through Correlation Coefficient (*CC*), Relative Bias (*RB*), and Root Mean Square Error (*RMSE*) between the satellite-estimated precipitation data and gauged rainfall observations. *CC* and *RB* describe the agreement between the satellite estimation and the reference. *RMSE* is used to measure the average error magnitude. *STD* reflects the degree of dispersion for individuals within the group:

1 $$CC = \frac{{\sum\nolimits_{i = 1}^{N} {(P_{i} - \overline{P})(S_{i} - \overline{S})} }}{{\sqrt {\sum\nolimits_{i = 1}^{N} {(P_{i} - \overline{P})^{2} \sum\nolimits_{i = 1}^{N} {(S_{i} - \overline{S})^{2} } } } }}$$

2 $$RB = \frac{{\sum\nolimits_{n = 1}^{N} {(S_{i} - P_{i} )} }}{{\sum\nolimits_{i = 1}^{n} {P_{i} } }}$$

3 $$RMSE = \sqrt {\frac{{\sum\nolimits_{i = 1}^{N} {(P_{i} - S_{i} )^{2} } }}{N}}$$

where *N* is the number of samples; $P_{i}$ and $\overline{P}_{i}$ denote the individual and mean gauged observation; $S_{i}$ and $\overline{S}_{i}$ denote the individual and mean satellite estimation.

#### Categorical statistical metrics

The skill in detecting precipitation for various satellite products is measured by Probability of Detection (*POD*), and False Alarm Rate (*FAR*). The *POD*, with a range from 0 to 1, indicates the ratio of the number of precipitation events correctly detected by satellite among all actual precipitation events. The *FAR* is the ratio of false alarming precipitation events to the total number of detected precipitation events, ranging from 0 to 1:

4 $$POD = \frac{a}{a + c}$$

5 $$FAR = \frac{b}{a + b}$$

where *a* denotes observed rainfall correctly detected, *b* denotes rainfall events detected, and *c* denotes observed rainfall events. The closer *POD* value is to 1 and the closer *FAR* value is to 0, the better skill in detecting precipitation and no-precipitation of the satellite dataset.

#### Hydrological model

As a distributed hydrological model, the VIC model has been widely used to assess and validate SPPs^48–50^. In this study, the VIC version 5 (VIC-5) model was set up at 0.25° × 0.25° spatial resolution grids in the YZRB. Information on soil parameters including soil properties and spatial distribution was retrieved from the International Geosphere Biosphere Program Data and Information System (IGBP-DIS)^51^. The vegetation parameters were obtained from Maryland 1 km global land cover products^52^ and the topography data was from Advanced Space borne Thermal Emission and Reflection Radiometer Global Digital Elevation Model (ASTER GDEM, 30 m)^53^. The main seven parameters were calibrated (Table1) through the Genetic Algorithm (GA), known as an effective parameters calibration method that can address the issues of premature convergence and permutation^54^. The Nash–Sutcliffe Efficient index (*NSE*)^55^ and Relative Error (*RE*) were used to evaluate model performance. A successive difference of *NSE* less than 0.001 is used as the stopping condition of the GA program to address the convergence issue^56^.

6 $$R{\text{E}} = \frac{{\sum\nolimits_{i = 1}^{N} {(Q_{sim,i} - Q_{obs,i} )} }}{{\sum\nolimits_{i = 1}^{N} {Q_{obs,i} } }}$$

7 $$NSE = 1 - \frac{{\sum\nolimits_{i = 1}^{N} {(Q_{obs,i} - Q_{sim,i} )^{2} } }}{{\sum\nolimits_{i = 1}^{N} {(Q_{obs,i} - \overline{Q}_{obs} )} }}$$

where $Q_{sim}$ and $Q_{obs}$ are the simulated and observed streamflow, respectively; $\overline{Q}_{obs}$ is the mean of the observed streamflow; *N* is the total number of days in the period.

**Table 1:** Description of the calibrated parameters of the VIC model in YZRB.

Parameter	Description	Unit	Range
B_inf	Variable infiltration curve parameter	–	0.01 ~ 1.00
Ds	Fraction of Ds_max_ where nonlinear baseflow begins	–	0.30 ~ 1.00
Ds_max_	Maximum velocity of baseflow	mm/day	10.00 ~ 50.00
Ws	Fraction of maximum soil moisture where nonlinear baseflow occurs	–	0.10 ~ 1.00
D1	Thickness of the first soil moisture layer	m	0.03 ~ 0.10
D2	Thickness of the second soil moisture layer	m	0.10 ~ 1.00
D3	Thickness of the third soil moisture layer	m	0.50 ~ 2.00

Despite the variations in accuracy and spatiotemporal resolutions, different satellite-based forcing data might exhibit similar runoff prediction skills after recalibrating the model using the respective precipitation products^19,56^. Therefore, in this study, two scenarios were proposed to simulate the runoff processes with diverse SPPs.

Scenario I (Rainfall-reconstruction-based calibration): (a) calibrate and validate the VIC model with the 10 km gridded precipitation dataset in streamflow simulation during 2003 ~ 2015; (b) replace the rainfall reconstruction forcing with precipitation from the five SPPs for independent validation from 2003 ~ 2015 using the reconstruction-calibrated model parameters.

Scenario II (Product-specific recalibration): Recalibrate VIC using the five SPPs, respectively, over the same calibration period and then simulate runoff using the specific parameter sets calibrated from different products over the same periods as Scenario I.

## Results

### Statistical performance of SPPs

**Figure 2:**
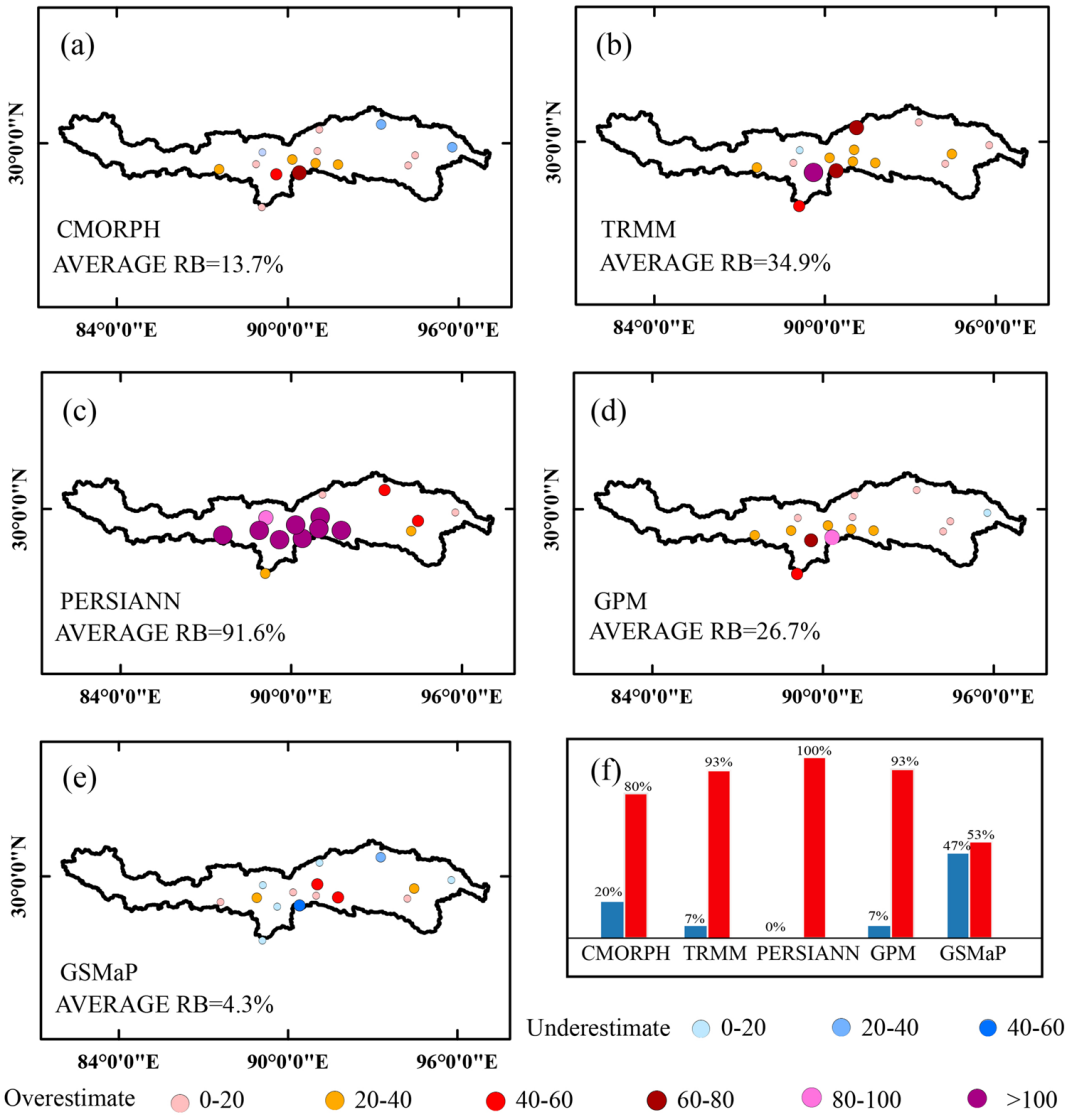
Relative Bias for (**a**) CMORPH, (**b**) TRMM, (**c**) PERSIANN, (**d**) GPM and (**e**) GSMaP and (**f**) fractions of underestimation (blue bar, %) and overestimation (red bar,%) stations against multi-year average gauged precipitation observations from 2003 to 2015 over YZRB.

**Figure 3:**
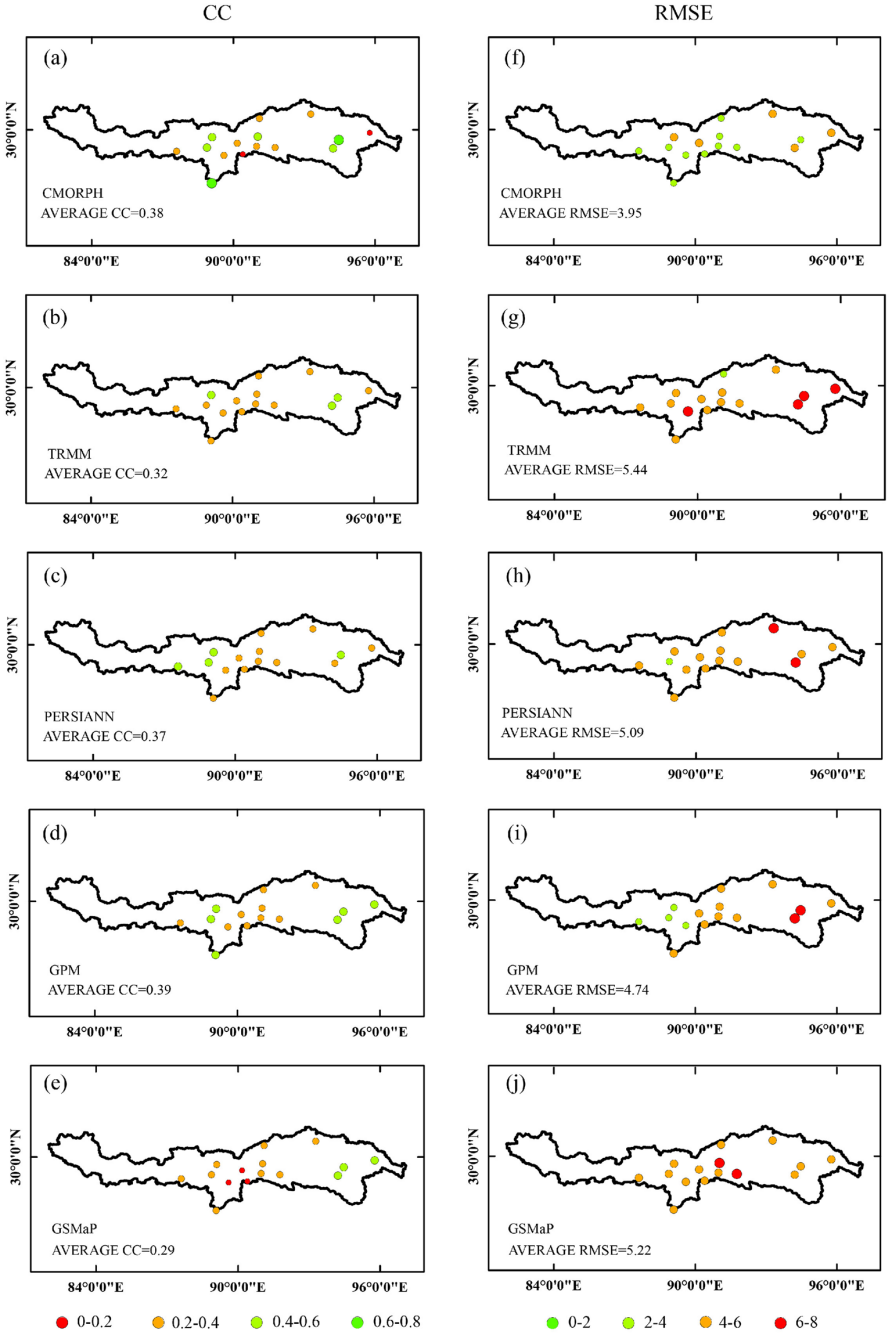
Spatial distribution of CC and RMSE between SPPs and gauged observations.

Figure 2 presented the spatial distribution of *RB* between five SPPs and gauged observations during the period from 2003 to 2015. As shown in Fig. 2, precipitation overestimation is indicated by warm colors and underestimation by cool colors; the larger the statistical metrics, the larger the circles (so are the same for Figs. 3 and 5). The four SPPs showed a general overestimation of precipitation from the perspective of *RB*, especially in the middle of the basin. For GSMaP, the overestimated and underestimated gauges are divided equally, which resulted in a low *RB* for the whole basin. Noticeably, PERSIANN tended to overestimate all gauges with an average *RB* of 92%, showing less skill for precipitation estimation compared to other datasets.

Moreover, the average *CC* and *RMSE* of diverse SPPs from 2003 ~ 2015 were also calculated at a gauge scale compared to rainfall observations (Fig. 3). Relatively higher *CC* and lower *RMSE* were found in the midstream area, while relatively lower *CC* and higher *RMSE* were detected in the downstream. This variation was probably due to the large amount of precipitation in downstream. CMORPH yield better accuracy in terms of *CC* and *RMSE* both in midstream and downstream over the YZRB.

**Figure 4:**
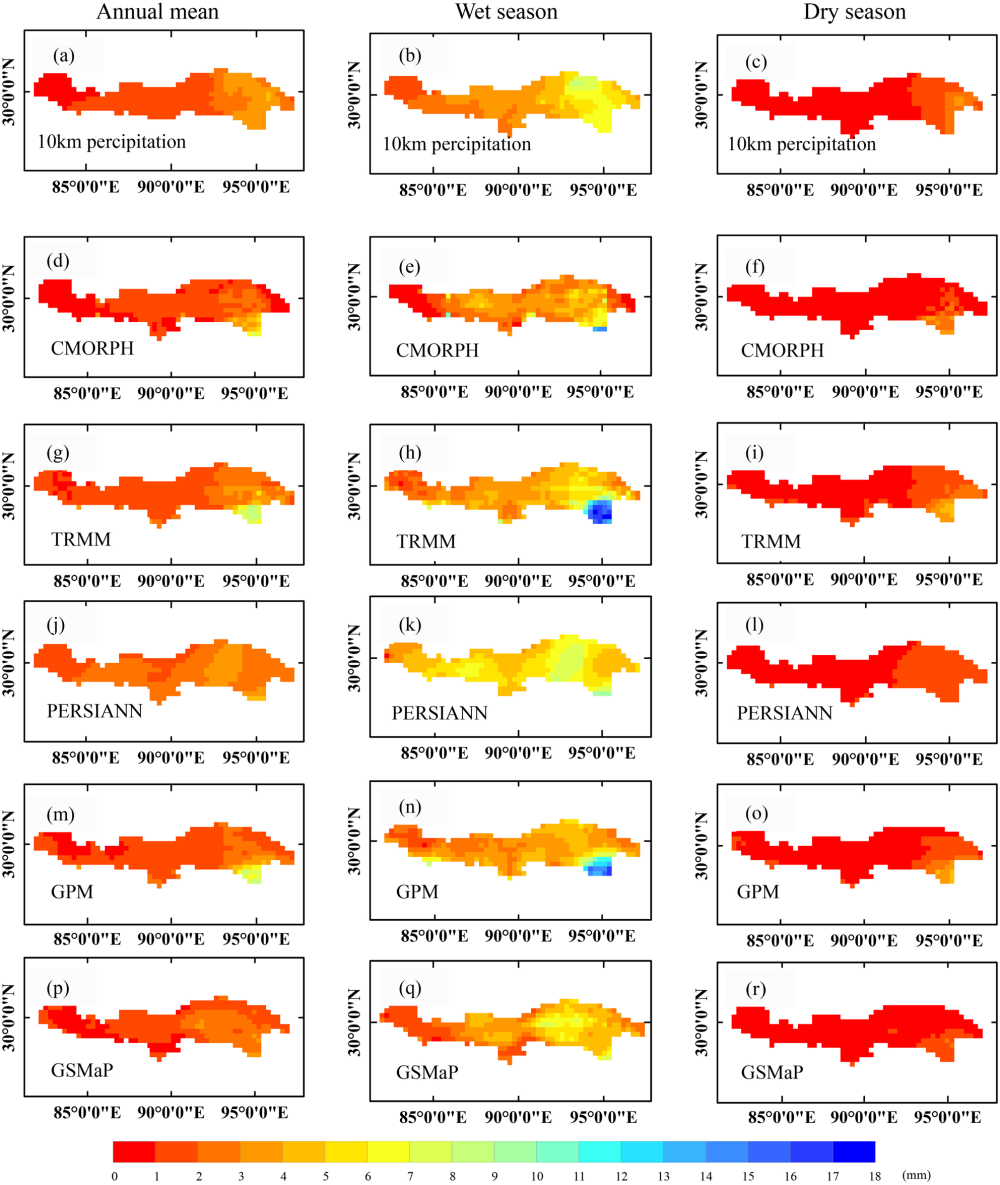
Spatial pattern of precipitation on multi-year timescale (left column), during wet seasons (middle column), and dry seasons in the YZRB during 2003–2015.

Figure 4 showed the seasonal differences as well as the multi-year average precipitation estimated using CMORPH, TRMM, PERSIANN, GPM, and GSMaP during 2003–2015. The results showed that all SPPs could generally capture the spatial precipitation pattern. Annual precipitation of 10 km precipitation data exhibited the east to west gradient, ranging from 3 ~ 4 mm/day in the east to less than 1 mm/day in the west (Fig. 4a). At the same time, the amplitude was significantly contrasting in the wet season because this period brings an ample amount of precipitation (Fig. 4b). Precipitation drew back to the southeast corners in the dry season and was less than 1 mm/day for most regions (Fig. 4c). CMORPH resembled the 10 km precipitation data in the annual and seasonal spatial patterns (Fig. 4d–f). However, the tendency of overestimation compared with the 10 km precipitation data was apparent, especially in the southeast region. In the wet and dry seasons, precipitation exceeded 14 mm/day and 5 mm/day, more prominent than 10 km precipitation data. TRMM estimation correlated well with 10 km precipitation data in the dry season, with precipitation decreasing from the southeast to the northwest of the YZRB ranging from 0 to 4 mm/day (Fig. 4f). However, disagreements were apparent in the southeast corner in the annual and wet period (Fig. 4g,h), where some precipitation patches didn't exist in the 10 km precipitation data. The PERSIANN estimation showed roughly consistent spatial variations with the 10 km precipitation data (Fig. 4j–l). The wet season didn’t appear a prominent precipitation patch in the southeast, while precipitation in most regions presented a higher range from 3 to 6 mm/day. In the annual and dry period, PERSIANN estimation demonstrated a basin-wide overestimation and an underestimation in the southern region, respectively. The GPM as the successor of TRMM exhibited identical good performance as TRMM compared with 10 km precipitation data (Fig. 4m–o), but there existed the precipitation patch in the corner of the southeast (Fig. 4m,n) in both the annual period and wet period. At the same time, in the dry season, the precipitation was underestimated in the northeast area. The GSMaP estimation showed a decreasing trend from east to west (Fig. 4p–r). Still, compared with 10 km precipitation data, the amount of precipitation was underestimated for the basin in the annual and seasonal period, especially in the northeast area.

**Figure 5:**
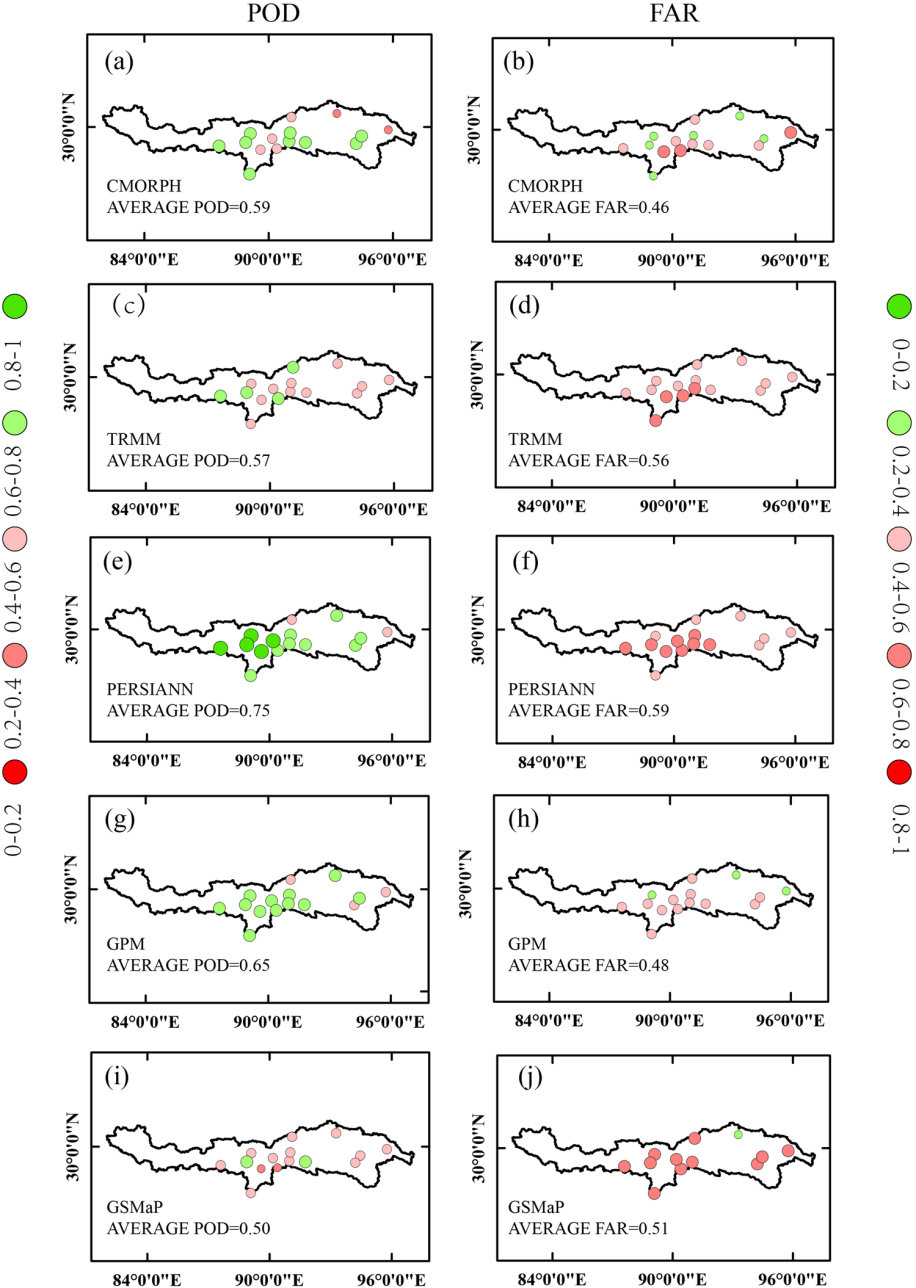
Spatial distribution of *POD* and *FAR* between SPPs and gauged observations.

The categorical statistical metrics (*POD*, *FAR*) at the daily time scale were shown in Fig. 5. The SPPs under this study showed an overall good performance, with PERSIANN showing the best performance with a *POD* of 0.75, followed by the GPM (*POD* = 0.65), CMORPH (*POD* = 0.59), TRMM (*POD* = 0.57), GSMaP (*POD* = 0.50). The regional *POD* analysis against gauged observations indicated the good capture ability in the middle of the basin for all SPPs. The PERSIANN showed the best performance with the *POD* range from 0.53 to 0.87. As seen in Fig. 5, major SPPs obtained better *POD* in the middle regions but showed a poor *POD* value in the down areas. This is because the POD values were higher in the drier regions and lower in the wetter areas. Conversely, all SPPs demonstrated lower FAR downstream and relatively higher *FAR* in the middle. Figure 5 indicated that CMORPH had the lowest *FAR* of 0.46, followed by the GPM (*FAR* = 0.48), GSMaP (*FAR* = 0.51), TRMM (*FAR* = 0.56), PERSIANN (*FAR* = 0.59). By contrast, the PERSIANN had the highest *POD* and highest *FAR*, probably caused by the overall overestimation of precipitation, indicating the inaccuracy of PERSIANN.

### Streamflow simulation

As we mentioned in “Hydrological model” section, two scenarios are adopted to evaluate and compare the five precipitation products against the gauged runoff observations on daily scale and different sets of calibrated parameters are shown in Table 2.

**Table 2:** Parameter calibration results in runoff simulation for VIC model under two scenarios.

Scenario	Products	B_inf	Ds	Dsmax	Ws	D1	D2	D3
I	CMORPH	0.57	0.37	36.66	0.90	0.10	0.28	1.94
	TRMM	0.57	0.37	36.66	0.90	0.10	0.28	1.94
	PERSIANN	0.57	0.37	36.66	0.90	0.10	0.28	1.94
	GPM	0.57	0.37	36.66	0.90	0.10	0.28	1.94
	GSMaP	0.57	0.37	36.66	0.90	0.10	0.28	1.94
II	CMORPH	0.84	0.79	18.92	0.89	0.04	0.16	1.49
	TRMM	0.56	0.40	17.81	0.96	0.06	0.57	1.43
	PERSIANN	0.07	0.51	14.73	0.81	0.05	0.74	1.74
	GPM	0.61	0.71	11.73	0.99	0.09	0.70	0.75
	GSMaP	0.39	0.63	14.88	0.58	0.05	0.94	0.93

**Figure 6:**
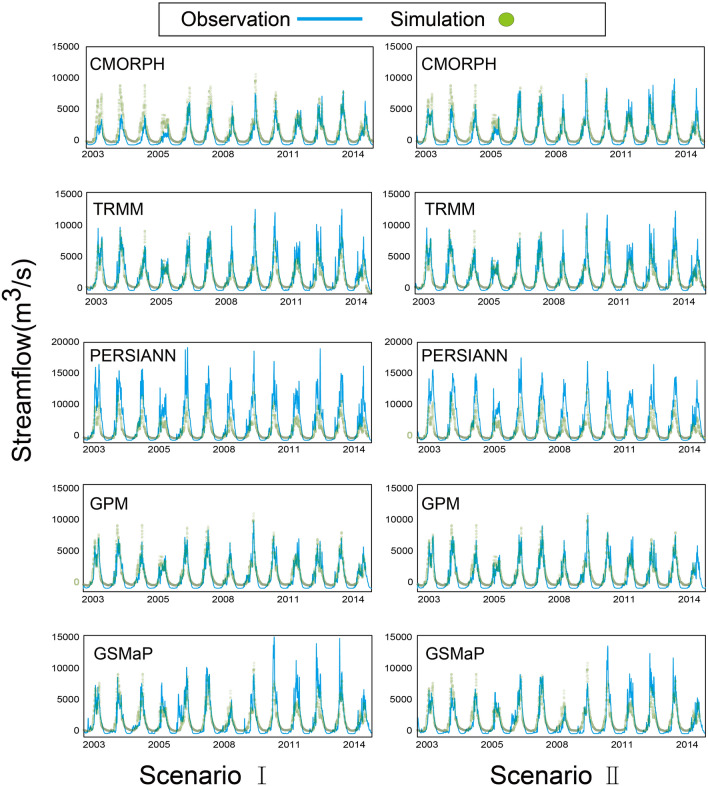
Results of daily streamflow at Nuxia under Scenario I and Scenario II.

Table 3 and Fig. 6 illustrated the contrasting accuracy and results of daily streamflow simulation at Nuxia under different scenarios. The results indicated that forced by various SPPs, the calibrated VIC model effectively captured the critical features of the observed hydrograph (Fig. 6). The GPM-driven VIC modeling had a daily *NSE* of 0.846 and *RE* of − 15%, and was shown to fit best with the observed streamflow amongst the five products (Table 3, Fig. 6). The PERSIANN-based runoff simulation systematically overestimated most of the streamflow series, with *NSE* of − 1.057 and *RE* of 71.8%. The GSMaP overestimated the streamflow by 3.1% from 2003 to 2015, probably due to the cancellation of precipitation bias in different periods. We found an underestimation before 2011 and an overestimation after it. The streamflow driven by TRMM exhibited satisfactory results with the *NSE* of 0.710 and *RE* of 11.0%, respectively (Table 3, Fig. 6). The streamflow driven by CMORPH had a trend of underestimation before 2007 but showed comparable quality with observations after that, resulting in an overall *NSE* of 0.693 and *RE* of − 36.3%.

**Table 3:** The VIC model accuracy in streamflow simulation driven by various precipitation data under two scenarios.

Scenario	Time series	Index	10 km precipitation data	CMORPH	TRMM	PERSIANN	GPM	GSMaP
I	2003 ~ 2010	*NSE*	0.87					
		*RE*	− 14.2					
	2011 ~ 2015	*NSE*	0.80					
		*RE*	− 9.5					
	2003 ~ 2015	*NSE*		0.69	0.71	− 1.06	0.85	0.49
		*RE*		− 36.3	11.0	71.8	− 15.0	3.1
II	2003 ~ 2010	*NSE*		0.77	0.85	− 0.70	0.86	0.73
		*RE*		− 32.1	− 4.2	64.5	− 19.6	− 19.2
	2011 ~ 2015	*NSE*		0.79	0.54	− 1.15	0.82	0.38
		*RE*		− 7.4	21.3	68.2	− 13.4	8.8

*NSE* indicates Nash–Sutcliffe efficiency, and *RE* is relative Error (%).

Further evaluation of the streamflow simulation potential of SPPs was conducted by calibrating the model with the corresponding satellite precipitation dataset in Scenario II. The calibration and validation periods were the same as that of Scenario I. Figure 6 showed the observed and simulated streamflow comparisons. The simulation performances from the three SPPs (TRMM, CMORPH, GSMaP) had improved after the individually calibrating, whereas the simulation from PERSIANN and GPM had minor changes. The simulation from the GPM had daily *NSE* of 0.86 and 0.82, and daily *RE* of − 19.6% and − 13.4% for the calibration and validation period, respectively, showing great potential in the hydrologic utility. The simulation from PERSIANN exhibited completely opposite results with *NSE* < 0. The discharge simulations from CMORPH had daily *CC* of 0.77 and 0.79 and daily *RE* of − 32.1% and − 7.4% for the calibration and validation periods. The discharge simulations from the TRMM and GSMaP showed different performances in the calibration and validation periods with *NSE* of 0.85 and 0.54, 0.73 and 0.38, respectively, probably due to the calibrated parameter's compensation in the calibration period.

**Figure 7:**
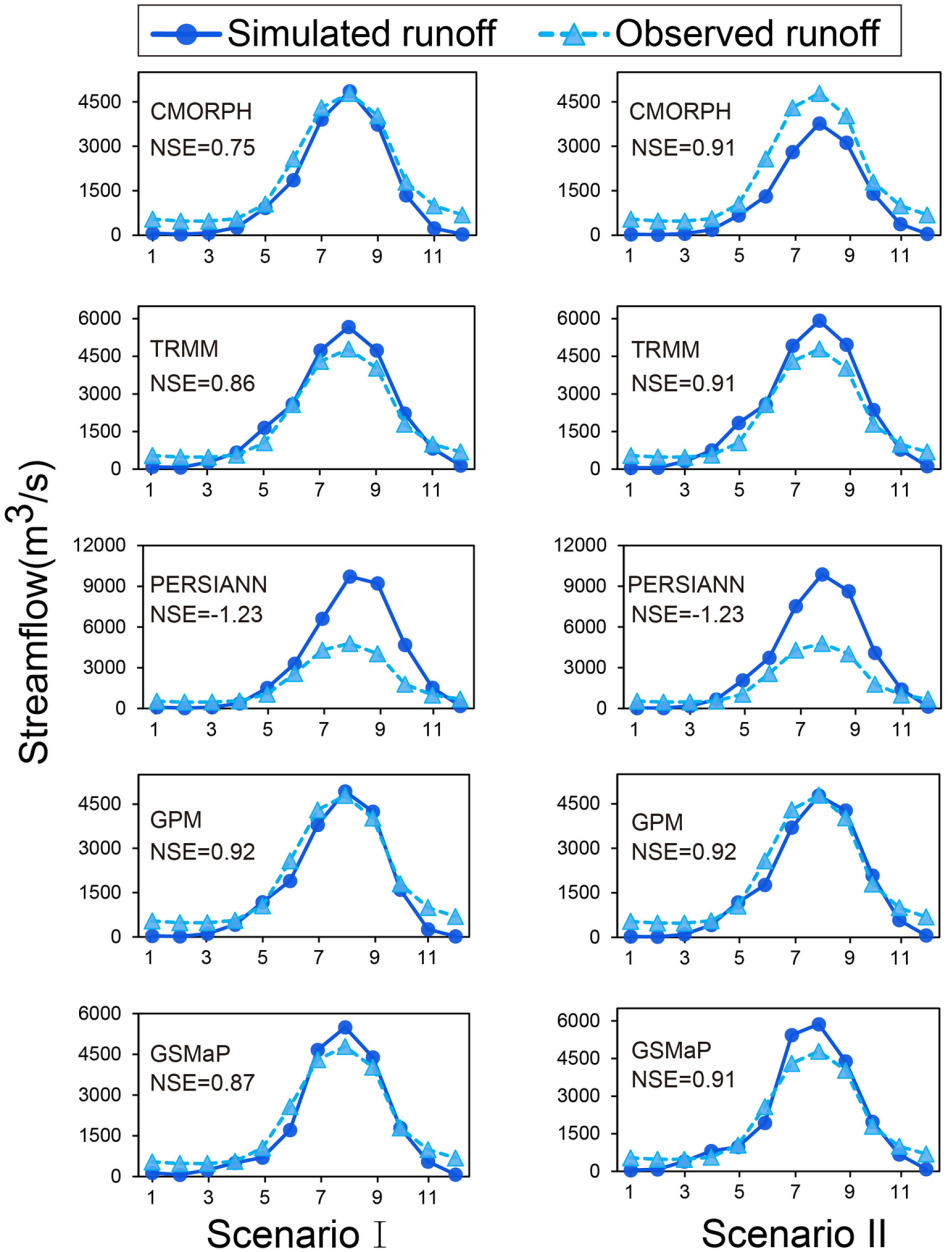
Multi-year average observed and simulated streamflow driven by different SPPs under Scenario I and Scenario II.

Figure 7 showed that all SPPs except PERSIANN could better describe the multi-year average trend in two Scenarios. Table 4 showed the *RE* between observed and simulated streamflow in dry (October to May) and wet (June to September) seasons. We can find that all SPPs performed better in the wet season with lower *RB* than in the dry season under both two Scenarios, and GPM performed best in the wet season, followed by GSMaP, TRMM, CMORPH, and PERSIANN. It was worth noting that TRMM performed better than its successor GPM in dry season simulation. It also indicated slight underestimation in the dry season for all SPPs expect PERSIANN against gauged observations in Fig. 7 and Table 4, which may be induced by the nature of the frozen soil algorithm and the poor ability to capture little rain of SPPs in dry season^2^.

**Table 4:** Relative Error statistics of simulated streamflow in YZRB in wet and dry seasons.

Scenario	Season	CMORPH	TRMM	PERSIANN	GPM	GSMaP
I	Wet	− 29.8	17.1	89.8	− 7.5	12.3
	Dry	− 57.5	− 4.9	31.1	− 32.3	− 23.5
II	Wet	− 8.5	12.8	83.5	− 5.3	3.6
	Dry	− 54.6	− 9.8	28.8	− 44.9	− 38.3

## Discussion and conclusion

### Discussion

There have been many studies attempting to assess the SPPs’ accuracy in scarce-gauged-data areas around the Third Pole, or the Qinghai-Tibet Plateau. Satellite precipitation assessment is particularly crucial to provide forcing inputs for basin-scale hydrological simulation. However, few studies conducted in YZRB have focused on the comprehensive evaluation of multi-satellite products^36,37^. In this study, multi-satellite precipitation products (GPM, TRMM, GSMaP, PERSIANN, CMORPH) are all incorporated with gauged observations, and were effectively assessed in terms of data reliability and hydrometeorological application potential via the well-calibrated VIC model over the YZRB. Results of the statistical analysis between the SPPs and gauged observation indicated that except for PERSIANN, other SPPs Generally, CMORPH, GPM and GSMaP present significant enhancement in rainfall estimations in comparison with TRMM and PERSIANN with lower *RMSE, RB, FAR* and higher *CC* and *POD* (Figs. 2, 3, and 5), despite the common misestimation that occurs in the southeast corner of the river basin (Fig. 4). Similarly, GPM and CMORPH have exhibited stronger potential in streamflow simulation than the others, indicated by higher *NSE* and lower *RE* (Fig. 6, Table 3). Ultimately, a correction process is highly needed for PERSIANN to use the local measurement systems to enhance the hydrological utility over YZRB. Results of the study may contribute to comprehensive assessing the skill and quality of rainfall estimates from multi-satellite products over YZRB.

The hydrological utility of satellite precipitation is closely associated with parameter estimations, input precipitation dataset, and model structure itself. To address the problem of differentiating spatial resolution of SPPs, the resampling method was conducted and facilitated the comparison among satellite datasets, despite that the resampling procedure could cause some errors inevitably and might further affect the accuracy of hydrological utility. Although GPM was resampled into a coarser resolution (0.25°), our study found significant improvements in GPM in both precipitation estimation and hydrological utility, similar to other studies in the TP^18^. Lots of studies have also documented that the GPM products, compared to their predecessor TRMM, are generally superior to TRMM in different area, such as the Xinjiang region^57^, Mainland China^58^, and Far-East Asia^59^. Nevertheless, it was found in our study that TRMM outperformed GPM in the dry season in runoff simulation, a noticeable property in these satellites that is worth studying. Liu and Yong^60^ pointed out that regions characterized by complex terrain and a rigid climate would still be challenging for the GPM and TRMM under current observing skills. Therefore, the complex terrain and the upward monsoon could also result in unexpected errors between SPPs and gauged observations over the YZRB. Furthermore, many researchers suggest that satellite precipitation estimations that incorporate rain gauge information perform better than satellite-only estimations^2,21^. In our study, five SPPs including GPM, TRMM, GSMaP, PERSIANN, and CMORPH were all incorporated with gauge observations, yet there is still a gap between the performance of these products and satisfactory estimation accuracy, different from previous studies that claimed high applicability after fusing SPP with gauge rainfall^2,16,17^. It may suggest that the algorithm used to incorporate rain gauge information need to be modified to adapt to the mountainous topography. Considering that almost no rain gauges are installed in the upper of the basin, more efforts should also be made to build denser rain gauges in these regions.

The uncertainties caused by parameters of hydrological modeling could also be influential on the SPP evaluation results. Ideally, the parameters are obtained by comparing the simulated value with the perfect value, later considered the best possible description of basin characters to run with different SPPs. We have introduced the widely proven high-quality rainfall products reconstructed by Sun and Su^39^ in Scenario I (Rainfall-reconstruction-based calibration), as the lack of gauged observations may hamper the evaluation of SPPs, especially in capturing extreme events in the historical period^27,61,62^. Moreover, we defined the search space of the VIC model parameters to be strictly within its physical field through the GA optimization procedure and converged the model to the optimal solution to decrease the parameter uncertainty^63^. However, calibrating the model with an identical parameter set tends to hamper the fairness of the evaluation of different SPPs, although it is widely used by the hydrological community, especially in gauged basins^64^ and product-specific recalibration might enhance the performances of hydrological modeling^56^. Results from our study indicated improved performances from CMORPH over calibration and validation periods and more promising estimates from other products over calibration periods when recalibrating the VIC model (Table 3). However, the contribution of glacial meltwater to runoff was not considered, which could introduce some uncertainty in the assessment of the SPPs, though the area covered by snow and ice in the YZRB is much smaller^65–67^. In the future, the evaluation of SPPs’ applicability in runoff simulation of YZRB could be enhanced by coupling VIC and glacier modules.

### Conclusion

By using the VIC model and statistical metrics, the five satellite precipitation products were evaluated in the YZRB on a daily scale. The main conclusions are as follows:

1. In general, all SPPs represented a similar rainfall pattern in the YZRB, demonstrating a decreasing trend from the east to the west. However, PERSIANN performed worst with an enormous overestimation in the basin. CMORPH performed better among SPPs, with slightly higher correlation and lower bias.
2. The GPM and CMORPH products exhibit comparable ability in streamflow simulations, indicating a great potential in the hydrological application. GPM performed best in daily streamflow simulation, followed by CMORPH, TRMM, GSMaP, and PERSIANN.
3. The GPM performed better for streamflow in the wet season than TRMM, while TRMM performed better in the dry season.

## Supplementary Information

Supplementary Information 1.

Supplementary Information 2.

Supplementary Information 3.

Supplementary Information 4.

Supplementary Information 5.

Supplementary Information 6.

Supplementary Information 7.

Supplementary Information 8.

Supplementary Information 9.

Supplementary Information 10.

Supplementary Information 11.

Supplementary Information 12.

Supplementary Information 13.

Supplementary Information 14.

## Data Availability

The streamflow data that support the findings of this study are available from the Tibet Hydrology and Water Resources Survey Bureau but restrictions apply to the availability of these data, which were used under license for the current study, and so are not publicly available. The streamflow data are however available from the authors upon reasonable request and with permission of the Tibet Hydrology and Water Resources Survey Bureau. The CMORPH, TRMM, PERSIANN, GPM-IMERG and GSMaP were obtained from ftp://ftp.cpc.ncep.noaa.gov/precip/, http://precip.gsfc.nasa.gov, http://fire.eng.uci.edu/PERSIANN/, https://gpm.nasa.gov, https://sharaku.eorc.jaxa.jp, respectively. The observed daily precipitation, maximum and minimum temperature, and average wind speed were obtained from http://data.cma.cn. The 10 km precipitation data was obtained from http://data.tpdc.ac.cn.
