# Supplementary material for: Evaluation of multiple satellite precipitation products and their potential utilities in the Yarlung Zangbo River Basin
Source: Sci Rep. 2022 Aug 3;12:13334. doi: 10.1038/s41598-022-17551-y (PMC9349210; doi:10.1038/s41598-022-17551-y)
Supplement: Supplementary file 14 — Supplementary Information 14. [file 41598_2022_17551_MOESM14_ESM.docx]

Data and material used were derived from public domain resources, and we submitted additional extracted satellite precipitation data and simulated runoff data.
